# AOH1996, an Inhibitor of PCNA, Sensitises Breast Cancer Cells to Cytotoxic Chemotherapy

**DOI:** 10.2147/BCTT.S636967

**Published:** 2026-09-17

**Authors:** Radhika Aiyappa-Maudsley, Thomas A Hughes

**Affiliations:** 1 School of Medicine, University of Leeds, Leeds, UK; 2 School of Health Sciences, York St John University, York, UK

**Keywords:** breast cancer, PCNA, DNA repair, small molecule inhibitors, cancer therapy, AOH1996

## Abstract

**Purpose:**

AOH1996 is an inhibitor of proliferating cell nuclear antigen (PCNA). We have assessed efficacies of AOH1996 as a therapeutic, alone and in combination with representative chemotherapies using breast cancer cells of luminal, HER2 positive, and triple negative subtypes.

**Methods:**

MCF-7, AU-565, MDA-MB-231, MDA-MB-468, and HCC1143 breast cancer cells were used. Effects of AOH1996, epirubicin, and/or docetaxel on cell proliferation/survival were assessed using MTT and clonogenic assays. DNA damage was evaluated by γ-H2AX immunofluorescence.

**Results:**

Single agent AOH1996 treatment reduced proliferation/survival in all cells tested in a dose- and time-dependent manner. Use of AOH1996 in combination with either epirubicin or docetaxel caused significant, consistent and substantial reductions in proliferation/survival that exceeded additive effects for the agents alone. For example, in clonogenic assays using MCF-7 cells, the lowest doses tested of epirubicin and AOH1996 caused inhibition of only 4% and 3% individually, while the combination caused significant inhibition by 34%, representing a 4.8-fold increase on the additive effect. Similarly, using AU-565 cells, docetaxel and AOH1996 caused 6% and 9% inhibition individually, while the combination caused 48%, representing a 3.2-fold increase on the additive effect. Nuclear γ-H2AX foci, indicative of double‑strand DNA damage, were quantified to investigate mechanisms. Combinations of AOH1996 with epirubicin or docetaxel consistently caused significant increases in foci that exceeded additive effects in every cell line, demonstrating that combinations were highly DNA-damaging.

**Conclusion:**

Based on our in vitro data, AOH1996 has potential as a breast cancer therapy and causes chemo-sensitisation in combination with chemotherapies standardly used clinically in primary breast cancer. Future in vivo studies are warranted.

## Introduction

Breast cancer is the most prevalent cancer in women, constituting approximately 15% of all new cancer cases.^1^ Despite the ever-expanding range of molecularly targeted therapies that are available for breast cancer therapy, traditional cytotoxic chemotherapy remains a critical component of primary treatment in most poor-prognosis cases, notably in the triple-negative and HER2 positive subtypes and in larger or locally advanced cancers of all molecular subtypes.^2–4^ However, chemoresistance, through a variety of mechanisms including drug efflux, reduced drug uptake, enhanced DNA repair, and suppression of apoptosis, is a barrier to effective treatment.^5^ Accordingly, metastasis-related mortality occurs in up to 30% of these cases, demonstrating the need for improvements to this already aggressive therapy.^6^,^7^

Proliferating cell nuclear antigen (PCNA) is a nuclear protein that helps maintain genomic stability by interacting with proteins involved in DNA replication and repair.^8^ It is an accessory protein that acts as a sliding clamp on DNA to facilitate docking of other proteins that are essential for genomic function.^9^ In breast cancer, PCNA is a marker of cellular proliferation, and increased levels are associated with poor outcomes,^10^,^11^ although this prognostic value has not been found consistently in the literature perhaps due to intra-tumoural heterogeneity in expression.^12^ Interestingly, PCNA appears to undergo post-transcriptional modifications in cancer cells to form the “caPCNA” isoform, which is expressed at low levels, if at all, in normal cells.^13^ Expression of caPCNA in malignant cells is associated with reduced DNA synthetic fidelity, indicating that cells expressing caPCNA have impaired DNA repair pathways. In the context of breast epithelial cells, caPCNA is detected in ductal carcinoma in situ, invasive, and metastatic cancer cells, but not in tissue from disease-free women or those with atypical ductal hyperplasia.^10^

Due to its over-expression in cancers and its essential role in cell survival, PCNA has been explored as a target for cancer therapy.^14^,^15^ AOH1996 is a small molecule inhibitor that specifically targets the cancer‑associated PCNA isoform, caPCNA;^16^ the chemical structure of AOH1996 is shown in Gu et al.^17^ Mechanistically, AOH1996 functions in a transcription‑dependent manner and enhances the interaction of PCNA with RPB1, the largest subunit of RNA polymerase II. This dissociates PCNA from active chromatin strands, resulting in accumulation of DNA double strand breaks.^17^ Notably, AOH1996 exhibits cancer cell specificity, showing minimal effects in normal cells even at six times higher concentrations than those effective in cancer cells.^17^ AOH1996 has shown promising results as a potential cancer therapeutic in pre-clinical models of acute myeloid leukemia^18^ and pancreatic cancer.^19^ It has also been tested in combination with the tyrosine kinase inhibitor osimertinib in lung cancer cells^20^ and with the chemotherapy agents topotecan, irinotecan, or cisplatin in neuroblastoma cells,^17^ showing at least additive effects and potentially synergy. Currently, AOH1996 is undergoing Phase I clinical trials in solid tumours (NCT05227326) and acute myeloid leukemia (NCT06763341). However, the anti-cancer and the chemo-sensitising effects of AOH1996 have not been evaluated in breast cancer cells. In the current study, we sought to assess cytotoxic effects on breast cancer cells of AOH1996, both as a monotherapy and in combination with chemotherapy agents that represent the classes most commonly used in primary breast cancer cases: namely, epirubicin (representative of anthracyclines) and docetaxel (representative of taxanes). These experiments were carried out in breast cancer cell lines in vitro, as an essential first step to determine potential for future pre-clinical and translational development of the agent in the breast cancer setting.

## Materials and Methods

### Cell Culture

Cell lines were initially procured from American Type Culture Collection (ATCC, Virginia, USA) and were validated for lack of mycoplasma contamination (MycoAlert; Lonza, Basel, Switzerland) and identity (short tandem repeat profiling; Source Bioscience, Nottingham, UK) prior to experimental use. MCF-7, MDA-MB-231 and MDA-MB-468 cells were cultured in Dulbecco’s Modified Eagle Media (DMEM) (Thermofisher Scientific, Massachusetts, USA). AU-565 and HCC1143 were grown in Roswell Park Memorial Institute (RPMI) (Merck, Darmstadt, Germany). All media were supplemented with 10% (v/v) FCS (Thermofisher Scientific, Massachusetts, USA) and 0.1% (v/v) penicillin–streptomycin (Thermofisher Scientific, Massachusetts, USA). All cells were incubated at 37°C in humidified air/5% CO_2_.

### Survival Assays

Cells were seeded in 96-well plates in complete media at 1000–3000 cells/well and incubated for 24h. Cells were treated with AOH1996 (Cambridge Bioscience, Cambridge, UK), epirubicin (Sigma Aldrich, Missouri, USA) and/or docetaxel (Sigma Aldrich, Missouri, USA) or were control treated for up to 48h in technical triplicates. Treatments were removed and replaced with fresh complete media lacking drugs before further incubation for 72h. MTT assays were carried out as previously reported.^21^ Briefly, 0.5mg/mL MTT solution (Thermofisher Scientific, Massachusetts, USA) was added to wells and incubated for 3h, forming formazan crystals. Solution was removed from wells and replaced by isopropanol and absorbance was read at 570nm, using a Mithras LB940 plate reader (Berthold, Bad Wildbad, Germany). For colony forming assays, cells were seeded in 6-well plates at 2×10^5^ - 3×10^5^ cells/well and incubated for 24h to allow attachment. Cells were then treated with AOH1996, epirubicin, and/or docetaxel or were control treated for 24h. Following treatment, cells were replated at low density (100–500 cells/well) in 6-well plates in technical replicates in media without drug for 14 days. Colonies were fixed, stained with 0.5% crystal violet (Sigma; St Louis, MO, USA), and counted as previously described.^22^ Colonies containing more than 50 cells were scored as a colony. Survival fraction was calculated as the number of colonies formed in treated cells relative to the untreated control, normalised by the plating efficiency.

### Immunofluorescence

Cells were seeded at 1.5×10^4^ - 2×10^4^ cells/well onto sterile coverslips in 24-well plates in technical triplicates and allowed to adhere overnight. The following day, cells were treated with AOH1996 (50nM) and/or epirubicin (5nM) and/or docetaxel (1nM) or were control treated for up to 48h. Coverslips were then washed in PBS and cells were fixed in 4% formaldehyde at room temperature for 20min. Following washing with PBS, cells were incubated in PBS, 0.3% Triton X-100 (Sigma, St Louis, MO, USA), 2% FCS (Thermofisher Scientific, Massachusetts, USA) for 30min to allow permeabilization. Cells were incubated overnight at 4°C with primary mouse monoclonal anti-γ-H2AX antibody (#AB26350 Abcam; Cambridge, UK), diluted 1:8000 in blocking buffer. After washing (3x) with washing buffer (PBS and 0.05% Tween-20), cells were treated with goat anti-mouse IgG AlexaFluor 488 Conjugate (A11029, Invitrogen, Oslo, Norway) at 1:1000 and incubated for 1h protected from light. Coverslips were then washed with wash buffer (3x) before mounting on Superfrost plus slides (Epredia, Michigan, USA) in Vectashield mounting media with DAPI (Vector laboratories, Newark, USA). Images were taken using a Zeiss Apotome wide-field fluorescent microscope (Zeiss, Oberkochen, Germany). Numbers of γ-H2AX foci per nucleus were counted using ImageJ software (NIH Freeware, Bethesda, MD, USA) on images of randomly selected fields of view under a ×63 objective; all the complete cells within multiple fields were included until a minimum of 100 cells had been counted for each treatment condition; the assessor was not blinded to the treatments but the automated counting software remains objective.

### Statistical Analyses

Experiments were performed in two or three independent replicates as indicated in each figure legend. Technical replicates within each biological experiment (for example technical replicate wells for survival assays, or separate nuclei on the same microscope slide for γ-H2AX assays) were used to determine a mean value for each independent experiment, and only these mean values were taken forward for statistical analyses using Prism v9.4.1 (GraphPad, San Diego, CA, USA). Survival analyses were analysed using non-linear regression, which also allowed estimation of IC_50_ doses, and foci counts were analysed using Wilcoxon signed rank tests. p<0.05 was taken to indicate significance.

## Results

### AOH1996 Causes Time- and Dose-Dependent Reductions in Proliferation/Survival of Breast Cancer Cells

**Figure 1:**
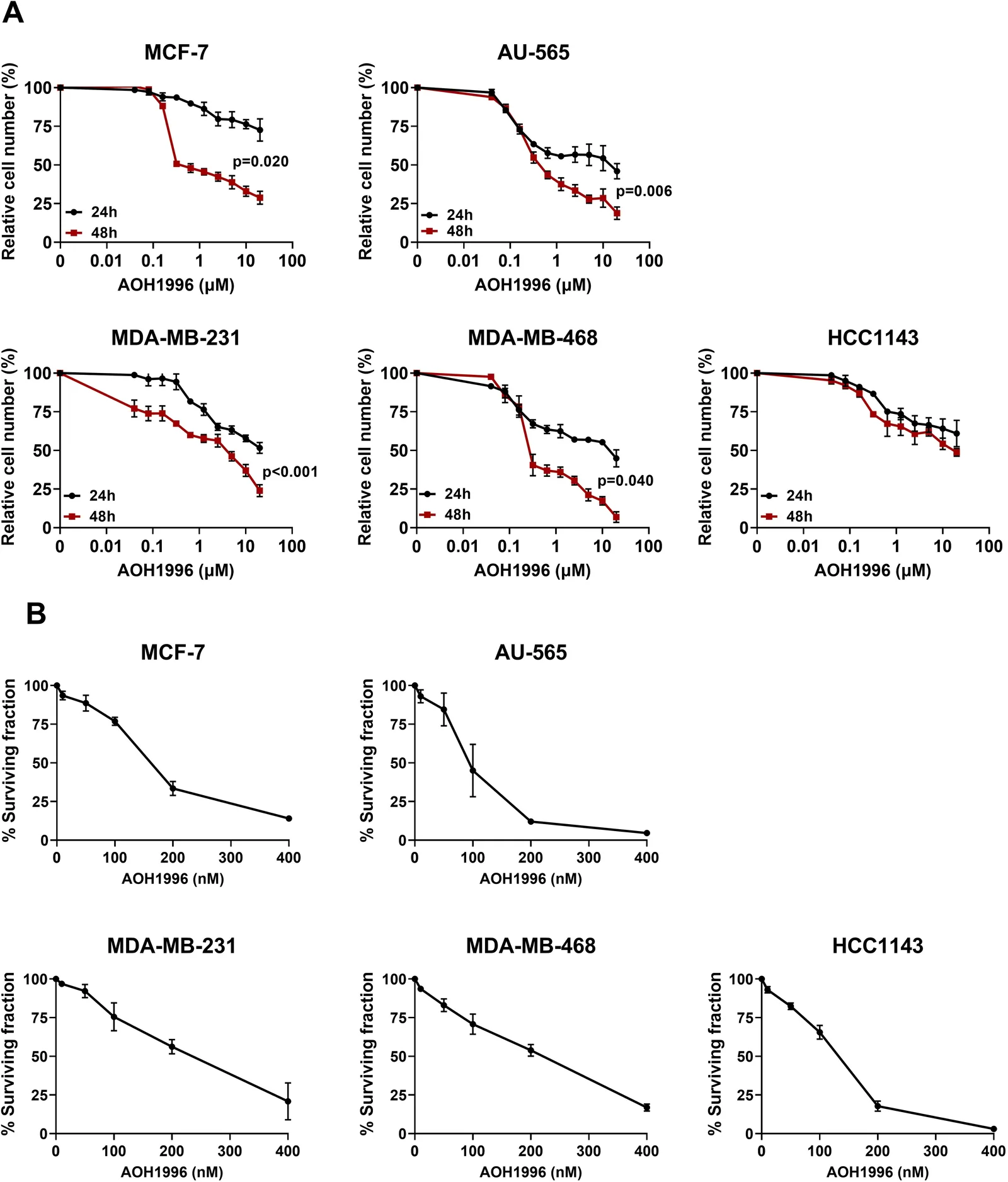
AOH1996 causes dose-dependent inhibition of proliferation/survival in breast cancer cell lines. Breast cancer cells as indicated were treated with varying concentrations of AOH1996, and proliferation was assessed (**A**) after 24h or 48h using MTT assays, or (**B**) after 24h treatment by clonogenic assays. Data are shown relative to untreated controls and represent means (± standard error) of three independent experiments. p-values were calculated from non-linear regression; p<0.05 was considered to be statistically significant.

A panel of breast cancer cell lines was assembled, encompassing those representing the different molecular subtypes: luminal (MCF-7), HER2 positive (AU-565), and triple negative cancers (MDA-MB-231, MDA-MB-468, HCC1147). Cells were treated with concentrations of AOH1996 up to 20µM for 24h or 48h, and cell proliferation/survival was assessed using MTT assays (Figure 1A). Treatment resulted in dose-dependent reductions in cell proliferation/survival in all cell lines, although there was considerable variation in sensitivity. IC_50_ doses ranged from 4.48μM to >20μM at 24h and 0.48μM to 14.02μM at 48h (Table 1). All cell lines showed greater inhibition at 48h compared to 24h, although this difference was comparatively small and not statistically significant in the most resistant cell line, HCC1143. While cell lines demonstrated differential sensitivity to treatment, this was not consistent across molecular subtypes, with triple negative lines showing both the most (HCC1143) and least resistance (MDA-MB-468). We also aimed to assess the implications of AOH1996 treatment on sustained proliferative ability, as this is more analogous to actual cancer treatment since the high doses required to kill cancer cells immediately in the previous assay are seldom achievable. The same panel of breast cancer cells was treated for 24h with doses of AOH1996 up to 400nM, and effects on clonogenic survival were assessed (Figure 1B). All lines demonstrated dose-dependent reductions in clonogenic survival, with IC_50_ doses ranging from 79.6nM to 227.7nM (Table 1). Surprisingly, the relative sensitivities of the cell lines differed in this assay as compared to MTT assays, with MDA-MB-231 being most resistant and AU-565 most sensitive, demonstrating the importance of using multiple assays. We concluded that AOH1996 is effective at reducing proliferation and survival across a range of breast cancer cell lines and has potential for further investigation as an anti-cancer agent in breast cancer.

**Table 1:** Tabulated Proliferation and Clonogenic IC_50_ for AOH1996

	Proliferation IC_50_ (μM) [CI]		Clonogenic IC_50_ (nM) [CI]
	24 hours	48 hours	
MCF-7	Not achieved	1.15 [0.71–1.88]	161.3 [120.2–217.2]
AU-565	4.48 [1.97–8.98]	0.66 [0.46–0.92]	79.6 [55.8–112.1]
MDA-MB-231	10.58 [7.88–14.31]	2.32 [1.34–3.96]	227.7 [175.1–298.6]
MDA-MB-468	5.35 [2.87–9.87]	0.48 [0.35–0.62]	187.4 [151.7–232.2]
HCC1143	Not achieved	14.02 [9.17–22.05]	106.1 [76.50–152.3]

**Abbreviations**: μM, micromolar; nM, nanomolar; CI, 95% confidence interval.

### AOH1996 Sensitises Breast Cancer Cells to Cytotoxic Chemotherapeutics

Next, we assessed impacts of AOH1996 in combination with the cytotoxic chemotherapy agents typically used to treat poor-prognosis primary breast cancers, namely a representative anthracycline, epirubicin, and a representative taxane, docetaxel. For these experiments, the panel of cell lines was reduced to only one line for each of the luminal (MCF-7), HER2 positive (AU-565), and triple negative subtypes (MDA-MB-231).

**Figure 2:**
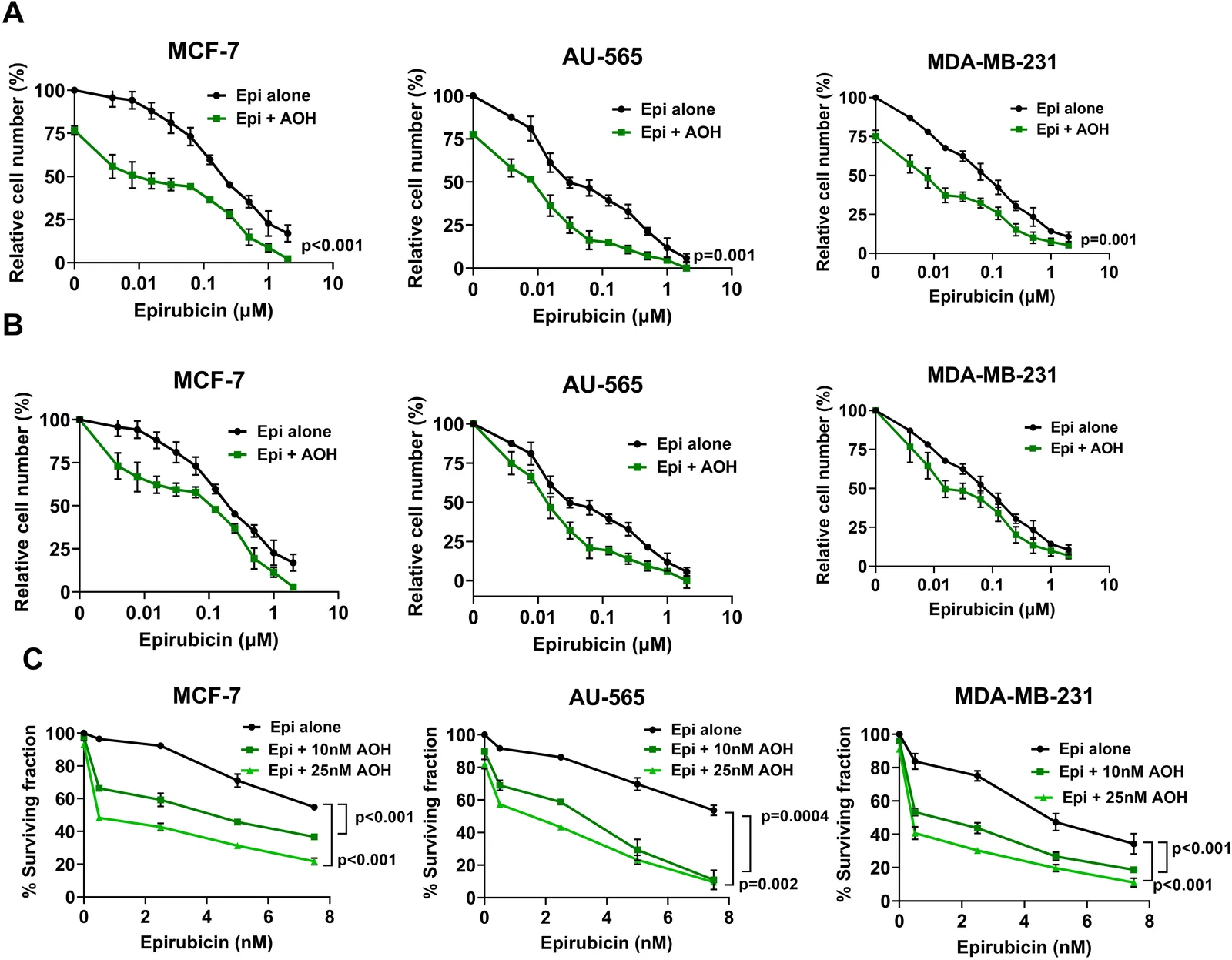
AOH1996 sensitises breast cancer cells to epirubicin. (**A**) Breast cancer cells as indicated were treated with varying concentrations of epirubicin alone or in combination with the IC25 dose of AOH1996 for each cell line (MCF-7 9.8μM, AU-565 0.07μM, MDA-MB-231 1.2μM). Proliferation/survival was assessed after 24h using MTT assays. (**B**) The same data are presented after survival for the combination treatments was expressed relative to the matched AOH1996 single agent treatment data, effectively removing the influence of AOH1996 alone. (**C**) Cells were treated for 24h with varying concentrations of epirubicin alone, or in combination with 10 or 25nM AOH1996. Survival was assessed by colony formation assays. Data are shown relative to controls treated with neither drug, and represent means (± standard error) of three independent experiments. Statistical analyses were conducted using non-linear regression.

Cells were treated for 24h with doses of epirubicin up to 2µM either alone or in combination with a relatively low dose of AOH1996 (selected as the IC_25_ dose from the 24h MTT assays data shown in Figure 1A). Cell proliferation/survival was assessed as previously, using MTT assays. As expected, combination treatments resulted in significantly increased inhibition of proliferation/survival compared to the single agent treatments (Figure 2A). Interestingly, when the single agent effect of AOH1996 was normalised away by expressing the data for the combination treatments relative to the AOH1996 alone values (Figure 2B), epirubicin consistently demonstrated enhanced efficacy in combination, suggesting that AOH1996 was acting as a chemosensitizer. As previously, effects were further assessed using clonogenic survival assays (Figure 2C). A range of epirubicin doses was used alone, or in combination with either 10nM or 25nM AOH1996. These AOH1996 doses were selected, based on the data in Figure 1B, as having only slight inhibitory effects alone (mostly >90% survival), thereby allowing a clear focus on whether these doses sensitised to epirubicin. As expected, epirubicin alone caused dose-dependent reductions in survival. Addition of these low doses of AOH1996 consistently caused a very substantial and significant enhancement of inhibition in the combinations. For example, in MCF7 cells the lowest doses of each agent caused only 4% and 3% inhibition individually, while the combination caused 34% - a more than 4.8-fold increase on the additive effect. Similarly, in MDA-MB-231 cells, this enhancement was 3.1-fold (single agents 4% and 13%; combination 53%). Using the same normalisation analysis as above, differences between epirubicin alone and combination treatments remained significant (Figure S1A), allowing estimation of apparent IC_50_ doses for epirubicin when used alone or in combination with AOH1996 (Table S1). These epirubicin doses were between 3.2- and 6.8-fold lower in the combination treatments compared to epirubicin alone, confirming that AOH1996 confers sensitisation to epirubicin.

**Figure 3:**
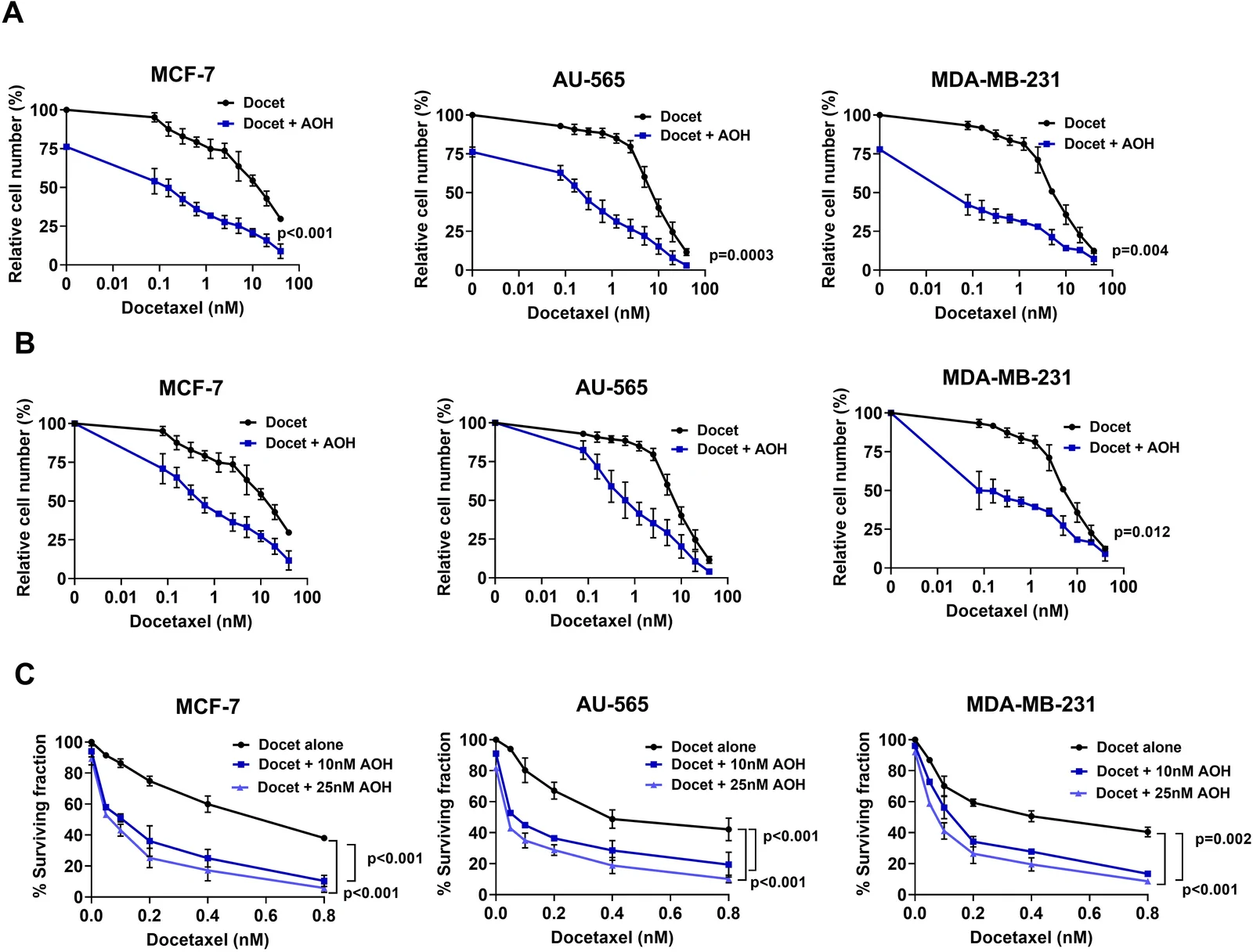
AOH1996 sensitises breast cancer cells to docetaxel. (**A**) Breast cancer cells as indicated were treated with varying concentrations of docetaxel or in combination with the IC25 dose of AOH1996. Proliferation/survival was assessed after 24h using MTT assays. (**B**) The same data are presented after survival for the combination treatments was expressed relative to its matched AOH1996 single agent treatment data, effectively removing the influence of AOH1996 alone. (**C**) Cells were treated for 24h with varying concentrations of docetaxel alone, or in combination with 10 or 25nM AOH1996. Survival was assessed by colony formation assays. Data are shown relative to controls treated with neither drug, and represent means (± standard error) of three independent experiments. Statistical analyses were conducted using non-linear regression.

The same experimental approach was taken to assess effects of the combination of docetaxel with AOH1996 (Figure 3). In MTT assays, this combination demonstrated significant increases in inhibition of proliferation/survival compared to docetaxel (Figure 3A) that were even greater than those seen with epirubicin. After normalisation, the increase in efficacy of docetaxel retained significance in MDA-MB-231 cells, demonstrating clear AOH1996-induced sensitisation (Figure 3B). In clonogenic assays, the combination treatment again showed inhibition that far exceeded additive effects (Figure 3C); for example, in MCF7 cells the lowest doses combined caused 43% inhibition, compared to the single agents 8% and 6% (a 3.1-fold increase) or in AU-565 cells 48% inhibition (single agents 6% and 9%; 3.2-fold increase). After normalisation (Figure S1B), apparent IC_50_ doses for docetaxel when used alone or in combination with AOH1996 dropped by between 2.8- and 7-fold (Table S2), again confirming that AOH1996 confers substantial sensitisation to docetaxel.

We concluded that low doses of AOH1996, that have only limited effects alone, caused significant sensitisation to the chemotherapeutics epirubicin or docetaxel.

### AOH1996 and Chemotherapy Agents Combine to Induce DNA Damage

**Figure 4:**
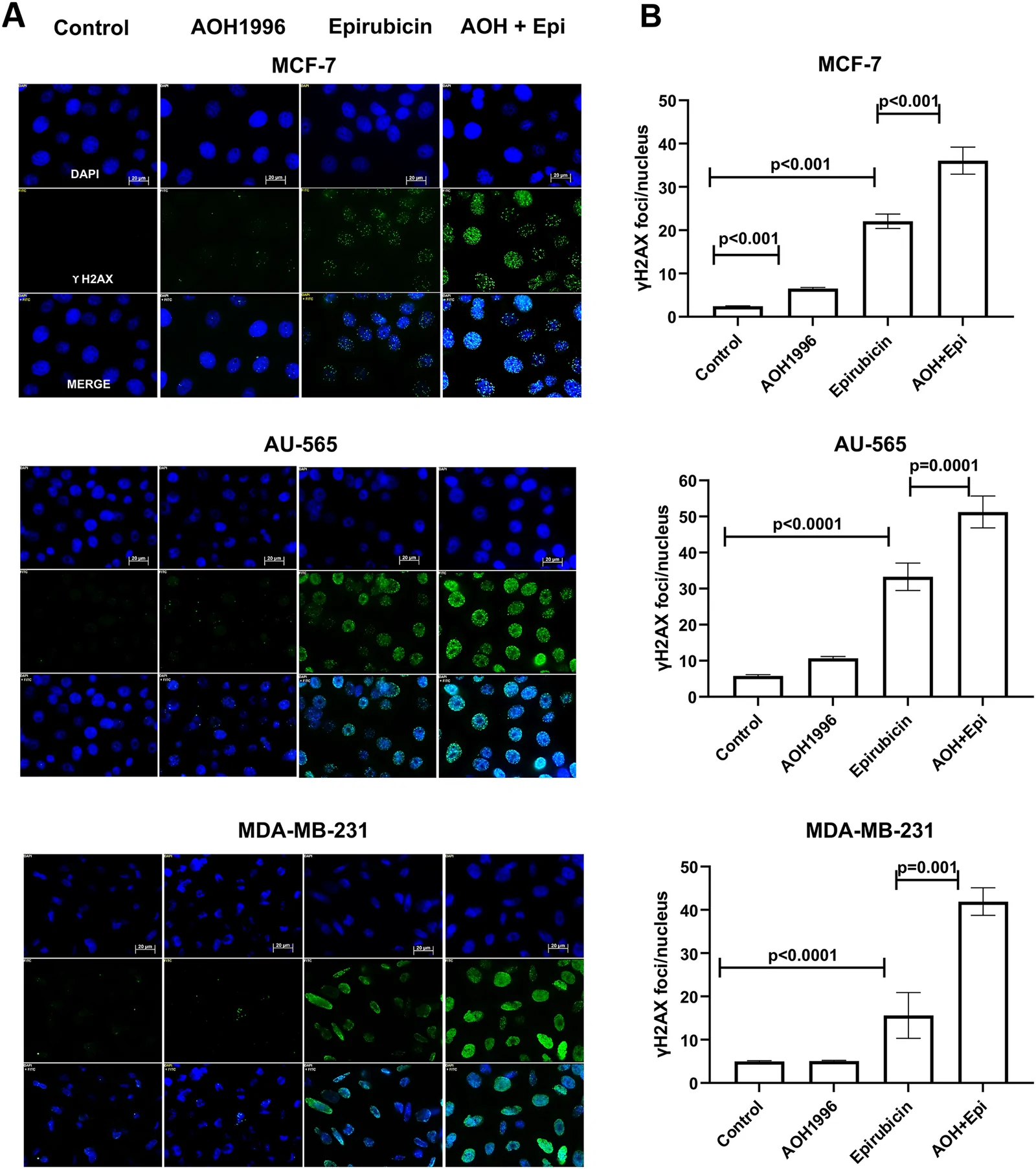
AOH1996 and epirubicin combine to cause dramatic increases in γ-H2AX foci in breast cancer cells. Breast cancer cells as indicated were control treated, or were treated with 50nM AOH1996, 5nM epirubicin, or the combination. Cells were stained using immunofluorescence to allow visualisation of γ-H2AX foci (green) and were counterstained with DAPI (cell nuclei, blue). Numbers of foci were counted in 100 cells, allowing a mean value to be calculated. (**A**) Representative images of γ-H2AX staining; (**B**) Graphical quantification of staining. Quantitative data represent mean foci (± SD) of two independent experiments. Statistical analyses were conducted using Wilcoxon signed rank tests. Images were obtaining used ×63 magnification. Scale bar represents 20μm.

**Figure 5:**
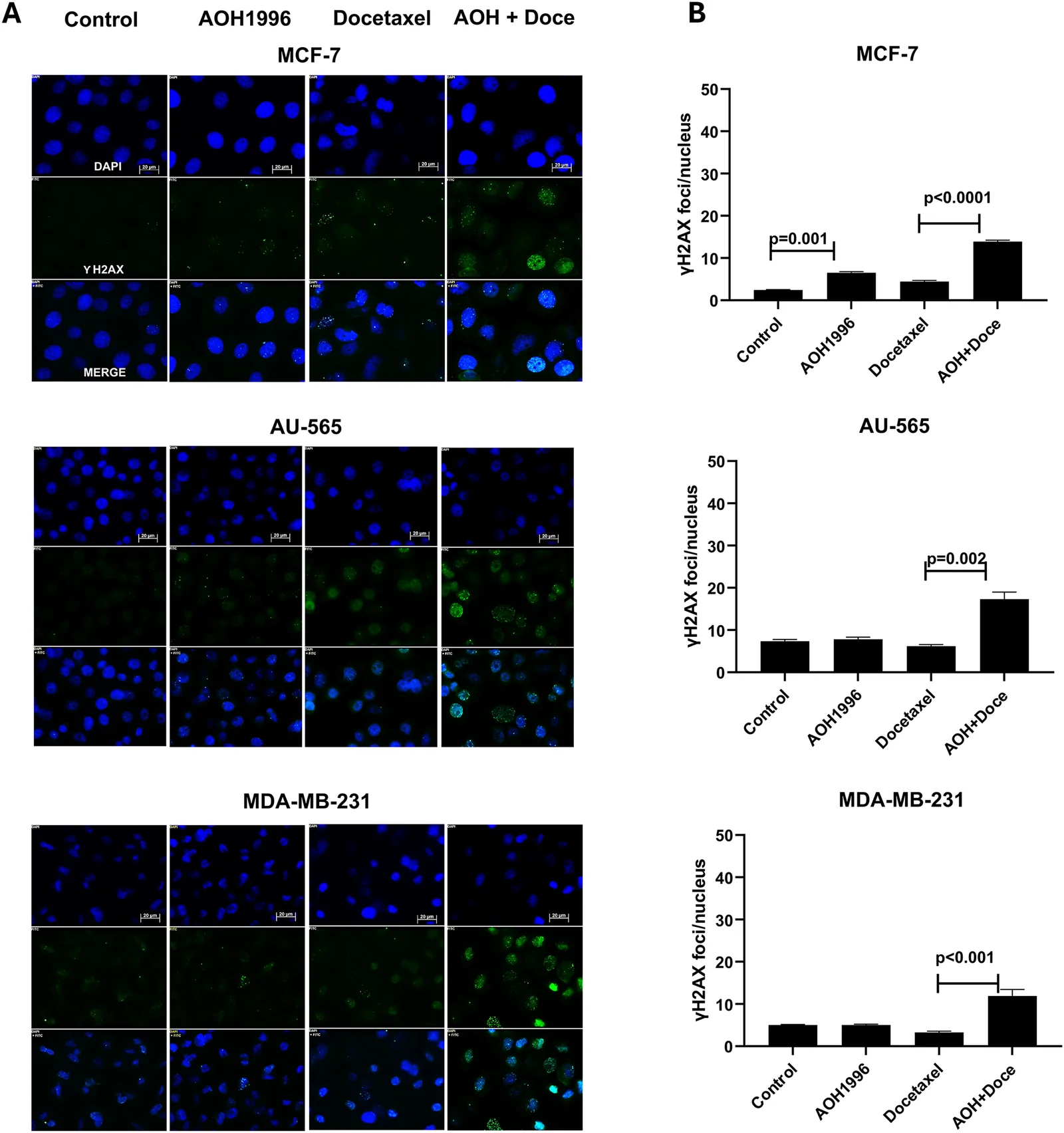
AOH1996 and docetaxel combine to cause dramatic increases in γ-H2AX foci in breast cancer cells. Breast cancer cells as indicated were control treated, or were treated with 50nM AOH1996, 1nM docetaxel, or the combination. Cells were stained using immunofluorescence to allow visualisation of γ-H2AX foci (green) and were counterstained with DAPI (cell nuclei, blue). Numbers of foci were counted in 100 cells, allowing a mean value to be calculated. (**A**) Representative images of γ-H2AX staining; (**B**) Graphical quantification of staining. Quantitative data represent mean foci (± SD) of two independent experiments. Statistical analyses were conducted using Wilcoxon signed rank tests. Images were obtaining used ×63 magnification. Scale bar represents 20μm.

AOH1996 binds to and inhibits the function of caPCNA, thereby creating transcription/replication conflict and subsequent DNA damage.^17^ Anthracyclines such as epirubicin also work partly by inducing DNA damage,^23^ while taxanes such as docetaxel are not classically regarded as DNA-damaging agents.^24^ Having noted that AOH1996 sensitises cells to both a DNA-damaging and a non-DNA-damaging chemotherapy agent, we were interested to investigate to what extent this interaction correlated with DNA-damage. Therefore, we quantified nuclear γ-H2AX foci, which are representative of double strand DNA breaks,^25^ in MCF-7, AU-565 and MDA-MD-231 cells after no treatment, or treatment with AOH1996 alone or in combination with epirubicin (Figure 4) or docetaxel (Figure 5).

As expected, low levels of γ-H2AX foci were detected in all cell lines when untreated, whereas epirubicin alone induced substantial and significant increases in foci numbers of up to 7-fold (AU-565 cells) (Figure 4). AOH1996 alone induced only a small, but significant, increase in γ-H2AX foci in MCF-7 cells, but not in the other lines. The combination treatments, however, induced substantial increases in foci that exceeded the additive effect of the single agents in every cell line. For example, in MDA-MB-231 cells AOH1996 alone induced no detectable increase in foci, while the combination induced formation of 2.7-fold more foci than epirubicin alone. Experiments with docetaxel reproduced data showing low basal levels of γ-H2AX foci in all cell lines, and the observation that AOH1996 induced significant foci in only MCF7 cells (Figure 5). Docetaxel alone, in accordance with its mechanism of action that does not involve explicit DNA damage, did not induce foci in any cell lines. However, the combination treatment was more interesting, as this was associated with a substantial and significant increases in foci in all 3 cell lines, which exceeded any additive effect (indeed in 2 of the 3 lines, neither individual agent induced any foci, so the theoretical additive effect would be zero). We concluded that AOH1996 and either epirubicin or docetaxel combine to induce increased levels γ-H2AX foci, which are consistent with increased levels of DNA damage; this represents a plausible mechanism of action for the sensitisation seen in Figures 2 and 3.

## Discussion

AOH1996 is an experimental, orally administered small-molecule inhibitor of PCNA that was identified recently.^17^ It was developed through modification of a parental compound, AOH1160, which demonstrated selective toxicity towards cancer cells by inhibiting DNA replication and homologous recombination DNA repair.^16^ Although effective in inhibiting PCNA, the unfavourable metabolic properties of AOH1160 prevented clinical advancement. In contrast, AOH1996 is a clinically compatible analogue with improved pharmacokinetics.^17^ It is interesting to note that a range of other potential therapeutic agents have been designed to target PCNA, including both small molecules and peptides.^26^ Overall, the general approach has been to design molecules that either inhibit or stabilise specific protein–protein interactions involving PCNA, thereby inhibiting elements of its downstream function. Examples include PCNA-I1^27^ which stabilises the trimeric form of PCNA, T2AA^28^ which binds PCNA competitively with DNA polymerase δ, and AOH1996 itself, which stabilises PCNA’s interaction with RPB1.^17^ Two agents have moved into clinical trials, with AOH1996 currently in Phase 1^15^ and ATX-101 advancing to Phase 2.^29^

We show here, for the first time, that AOH1996 is effective as a monotherapy at reducing proliferation/survival in a range of breast cancer cell types, spanning the molecular subtypes used to classify breast cancers (Figure 1). We used two key assays. The first assesses immediate cell survival, using MTT assays that determine cellular oxidoreductase and mitochondrial dehydrogenase activities as a surrogate for viable cell number. These assays were used with short-term drug treatments and therefore require relative high doses to induce rapid cell death. The second assay, colony forming assays, determines long-term proliferative survival of cancer cells after short-term treatment. This second assay is sensitive to terminal cellular damage that does not cause immediate cell death but nevertheless halts successful proliferation; it consequently requires much lower doses and is more reflective of some aspects of clinical cancer treatments where the aim is to stop cancer growth while using the lowest effective dose, thereby reducing side-effects. It should be noted that despite the facts that these assays measure different endpoints and used different doses of the agents and that the cell lines had varied intrinsic sensitivities to AOH1996 (Figure 1), similar chemosensitisation was seen in both assays and for all cell lines tested (Figures 2 and 3).

A key interest was how AOH1996 would combine with existing breast cancer therapeutics, since this is the most likely clinical translational pathway. PCNA inhibitors, including AOH1996, have been shown to stall replication forks and/or inhibit DNA repair and thereby lead to increased levels of DNA damage.^19^,^28^,^30^ Therefore, we examined the effects of AOH1996 in combination with traditional cytotoxic chemotherapy agents, which also typically act at least in part through inducing DNA damage. Anthracyclines and taxanes are the main chemotherapy classes used in primary breast cancer^31^ and have also not previously been tested in combination with AOH1996, therefore we examined the representative examples epirubicin and docetaxel. We found that the combination treatments displayed potent inhibition of cell proliferation/survival that far outstripped the additive effect of the agents separately (Figures 2 and 3). This result is in accordance with similar sensitisation seen for AOH1996 in combination with topotecan, irinotecan or cisplatin,^17^ and for other PCNA inhibitors in combination with cisplatin or radiotherapy,^30^ all of which are DNA-damaging anti-cancer therapies. While sensitisation to DNA-damaging agent epirubicin was perhaps expected in this context, the result with docetaxel was surprising since taxanes primarily act by inducing mitotic stalling and subsequent apoptosis,^24^ as opposed to through DNA damage. Consequently, we assessed DNA damage in the form of γ-H2AX foci, as markers of DNA double stand breaks, in the cells under the various treatments (Figures 4 and 5) to gain mechanistic insight into the interaction between the agents. At the dose used, AOH1996 alone induced only low levels of γ-H2AX foci and in only one cell line, while as expected epirubicin was highly DNA-damaging and docetaxel induced no damage. As was seen for cell survival, the combinations gave much higher levels than either agent alone of γ-H2AX foci, which were consistent with increased DNA damage (Figures 4 and 5). This result was especially striking for docetaxel; for example, in two cell lines neither agent induced any detectable foci alone, yet the combination induced significant levels of foci. One hypothesis is that this interaction may relate to the fact that both agents have been reported to cause mitochondrial disfunction leading to increases in levels of reactive oxygen species,^18^,^32^ which can be DNA-damaging when they exceed the capacity of endogenous antioxidants.^33^ In any event, the potential increases in DNA damage provide a plausible explanation for the sensitising activity of AOH1996 seen with both the anthracycline and the taxane. It should be noted that our findings are limited to in vitro analysis with cancer cell lines, and we have not carried out analysis in non-cancer lines or the in vivo validation that would often be needed before considering further clinical development. However, AOH1996 is already under clinical investigation as a single agent in phase 1 in solid cancers, and in combination with tyrosine kinase inhibitors (ClinicalTrials.gov registration NCT05227326), therefore, it is interesting to consider that clinical data concerning combinations involving AOH1996 may outpace published pre-clinical testing. We recommend that clinical testing of AOH1996 in combination with cytotoxic chemotherapy may be worthwhile.

In conclusion, we find that AOH1996 has potential for further investigation as a therapy in breast cancer both as a monotherapy and in combination with standard-of-care chemotherapy. In particular, the combination treatments were characterised by sensitisation to chemotherapy agents which correlated with enhanced DNA damage.

## Data Availability

All data are shown within the paper or are available from the corresponding author, Thomas A Hughes, on request.
